# The Antibacterial Effectiveness of Citrullus lanatus-Mediated Stannous Nanoparticles on Streptococcus mutans

**DOI:** 10.7759/cureus.45504

**Published:** 2023-09-18

**Authors:** Shruthi Rajagopal, Surendar Sugumaran

**Affiliations:** 1 Department of Conservative Dentistry and Endodontics, Saveetha Dental College, Saveetha Institute of Medical and Technical Sciences, Saveetha University, Chennai, IND

**Keywords:** green nanosynthesis, streptococcus mutans, nanoparticles, stannous nanoparticles, antimicrobial

## Abstract

Introduction

Dental caries is a prevalent oral health issue caused by the colonization of *Streptococcus mutans* in the oral cavity. *Citrullus lanatus*, commonly known as watermelon, is rich in bioactive compounds that possess antibacterial potential. In this study, we aimed to synthesize stannous chloride (SnCl_2_) nanoparticles (NPs) mediated by *Citrullus lanatus* extract and investigate their antibacterial effectiveness against *Streptococcus mutans*.

Materials and method

Stannous nanoparticles (SnNPs) synthesized by the green method were achieved by using the watermelon extract. Dilute stannous chloride solution was obtained by adding 0.45 g of stannous (Sn) chloride (Cl) powder to 60 mL of water, which was subjected to an orbital shaker with the watermelon extract. The nanoparticles obtained were subjected to characterization using antimicrobial testing, Fourier transform infrared (FTIR) spectroscopy, energy-dispersive X-ray (EDAX) analysis, and scanning electron microscopy (SEM). Agar well diffusion method was used against specific strains of *S. aureus*, *S. mutans*, and *Escherichia coli*.

Results

The novel nanoparticles demonstrated promising antibacterial activity against *S. mutans* providing 10 mm of inhibitory action.

Conclusion

Due to its abundance of naturally occurring bioactive chemicals and improved efficacy against *S. mutans*, watermelon extract can be utilized to create stannous nanoparticles as opposed to the use of toxic chemicals. They can also be employed as oral administration systems.

## Introduction

In recent times, the rapid increase of microbial drug resistance to modern antibiotics, along with the increasing propensity of microbial infections and the rapid evolution through mutation, calls for the creation or modification of antimicrobial substances, as well as alternative therapies [1]. A platform to combat bacterial mutation has recently been suggested by advanced research in nanotechnology, which has produced nanoscale items with notable antibacterial activities against multidrug-resistant infections. Metal nanoparticles' (NPs) distinct mechanism of action makes them the most effective among the most cutting-edge nanotechnological applications [2].

Certain metals have actively been used as antimicrobial agents and have strong scientific evidence, based on the evidence of the lethal effects of essential metals in excess doses and nonessential metals even at very minute doses [3]. Some antibiotics, such as bacitracin, bleomycin, streptonigrin, and albomycin, have metal ions tightly bound to the antibiotic structure that control the biocidal action. Other antibiotics, such as tetracyclines, aureolic acids, and quinolones, have metal ions attached to the antibiotic molecule that do not significantly alter the antibiotic structure but increase activity.

The creation of metal nanoparticles sparked a great deal of curiosity among scientists and nanotechnologists because of their microbicidal properties. With diverse metals such as silver, copper, zinc, titanium, stannous (Sn), and gold, attempts have been made for the synthesis of green synthetic nanoscale objects [4]. Other greenly synthesized metals, such as gold, find numerous other applications in a variety of engineering and technological fields; in contrast, the silver nanoparticles (AgNPs) synthesized by the green method find potential applications in the biomedical field, particularly in the development of antimicrobials (AgNPs) [5].

Due to their powerful antimicrobial effects against both gram-positive and gram-negative bacteria, viruses, and other eukaryotic microorganisms, stannous nanoscale particles with a high surface-area-to-volume ratio (size below 100 nm) are of particular interest when compared to other metals in their nanoform [6]. Tin oxide (SnO_2_) and tin oxide nanoparticles (SnO_2_NPs) have emerged as the most significant metal oxide nanoparticles due to a variety of factors, including their low operating temperature and cytotoxic, antibacterial, antioxidant, and biocompatibility properties [7]. The utilization of transition metal nanoparticles has grown in popularity due to their potential for photosynthesis, as well as their biocompatibility, low toxicity, and ability to be produced and used in an environmentally friendly manner.

Traditionally, the key challenge in nanoparticle synthesis is the development of environmentally friendly and sustainable methods. Conventional synthesis approaches often involve the use of hazardous chemicals and high-energy processes, which conversely affect the environment, not to mention human physical health. To provide solution to the abovementioned issues, researchers have turned their attention toward green synthesis, a sustainable approach that utilizes natural sources, such as plant extracts, as reducing and stabilizing agents for nanoparticle synthesis [8].

*Citrullus lanatus*, commonly known as watermelon, is a fruit widely consumed for its refreshing taste and nutritional benefits. Watermelon not only is rich in vitamins, minerals, and antioxidants but also contains bioactive compounds with potential biological properties. These compounds, such as polyphenols, flavonoids, and terpenoids, have been reported to possess antibacterial, antifungal, and antioxidant activities. Consequently, watermelon extract has drawn a lot of interest as a natural source for environmentally friendly nanoparticle manufacturing [9].

The utilization of watermelon extract in nanoparticle synthesis offers several advantages. Firstly, watermelon extract serves as an eco-friendly and cost-effective alternative to conventional reducing and stabilizing agents. The bioactive compounds present in the extract can act as reducing agents, facilitating the conversion of metal ions into nanoparticles. Secondly, these compounds can also serve as stabilizing agents, preventing the agglomeration and subsequent loss of nanoparticle activity. Moreover, the biological properties of watermelon extract contribute to the potential antimicrobial efficacy of the synthesized nanoparticles. The antibacterial activity of watermelon extract has been demonstrated against various pathogenic bacteria, including *Escherichia coli*, certain strains of *Streptococcus*, and *Pseudomonas aeruginosa*. These antimicrobial properties can be attributed to the presence of bioactive compounds that disrupt bacterial cell membranes, inhibit enzyme activity, and interfere with bacterial adhesion and biofilm formation. By incorporating watermelon extract in nanoparticle synthesis, the resulting nanoparticles may inherit these biological properties, thereby enhancing their antimicrobial effectiveness against target pathogens such as *Streptococcus mutans* [10].

The use of toxic precursor chemicals, such as sodium borohydride, potassium bitartrate, methoxypolyethylene glycol, and hydrazine; toxic solvents, such as sodium dodecyl benzyl sulphate; and toxic by-products is an additional drawback of traditional (physical and chemical) methods of nanoparticle synthesis [11]. As science develops, more environmentally benign methods for making metal nanoparticles are discovered. Here, metal salts are transformed into nanoparticles using a variety of biologically derived agents (derivatives of bacterial and fungal substances). These substances function as in vitro capping and reducing substances. Utilizing natural substances, such as phytochemicals, has been suggested as a potential cancer management technique. These substances have a wide range of biological activity, are inexpensive, and have few undesirable side effects.

In this study, we aimed to synthesize and characterize stannous chloride (SnCl_2_) nanoparticles mediated by *Citrullus lanatus* extract and evaluate their antimicrobial efficacy against *Streptococcus mutans*.

## Materials and methods

*Citrullus lanatus* extract preparation

**Figure 1:**
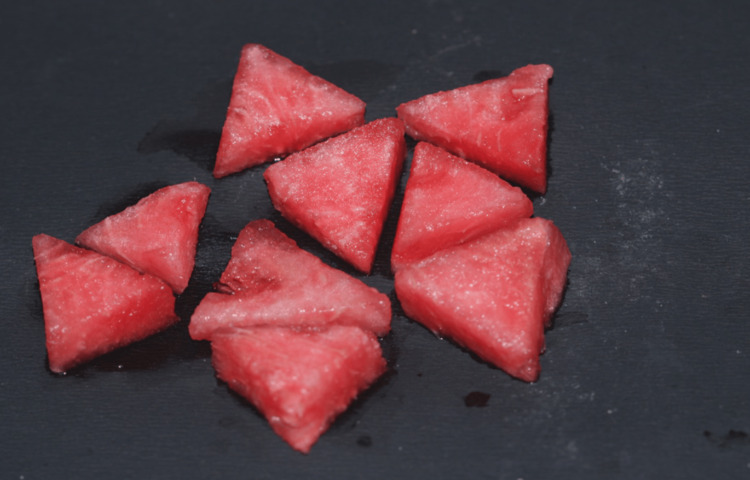
Native variety of Citrullus lanatus

Watermelon seeds and red flesh were obtained from the native variety of watermelon as it has the highest phenolic content. After washing thoroughly with distilled water, smaller pieces of the fruit were obtained that were subjected to sunlight for seven days to completely dry the fruit as seen in Figure 1.

**Figure 2:**
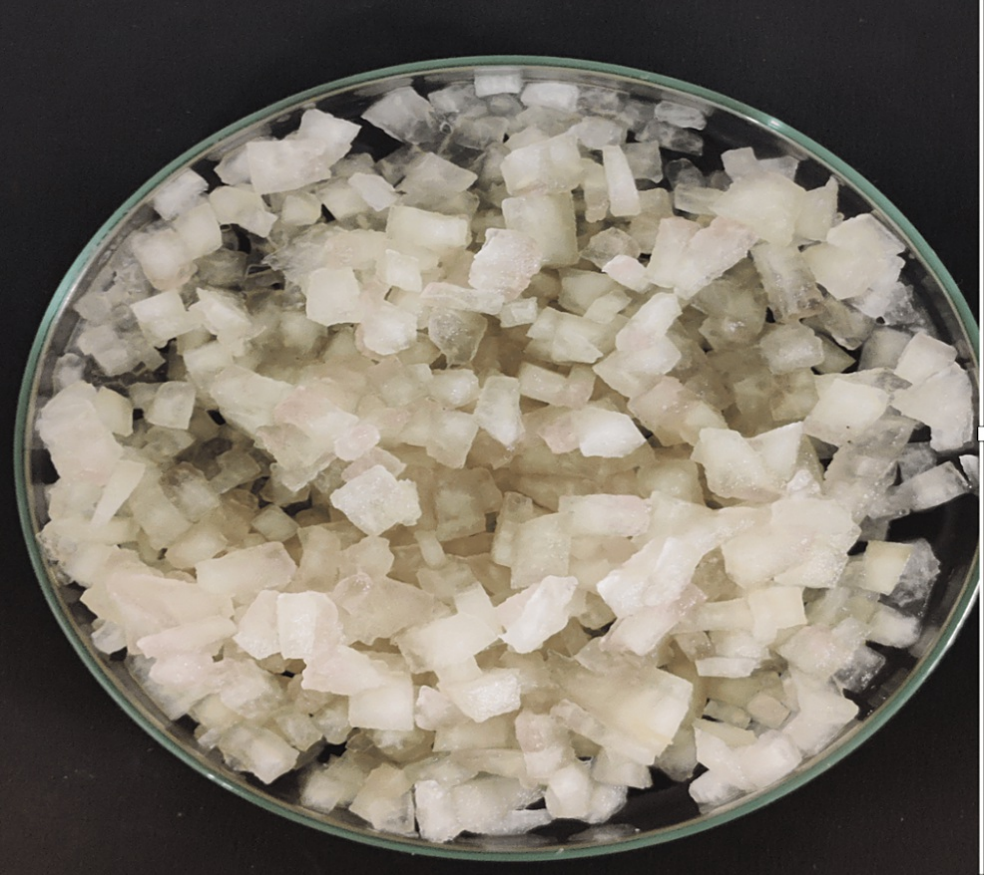
Extracted watermelon rind

**Figure 3:**
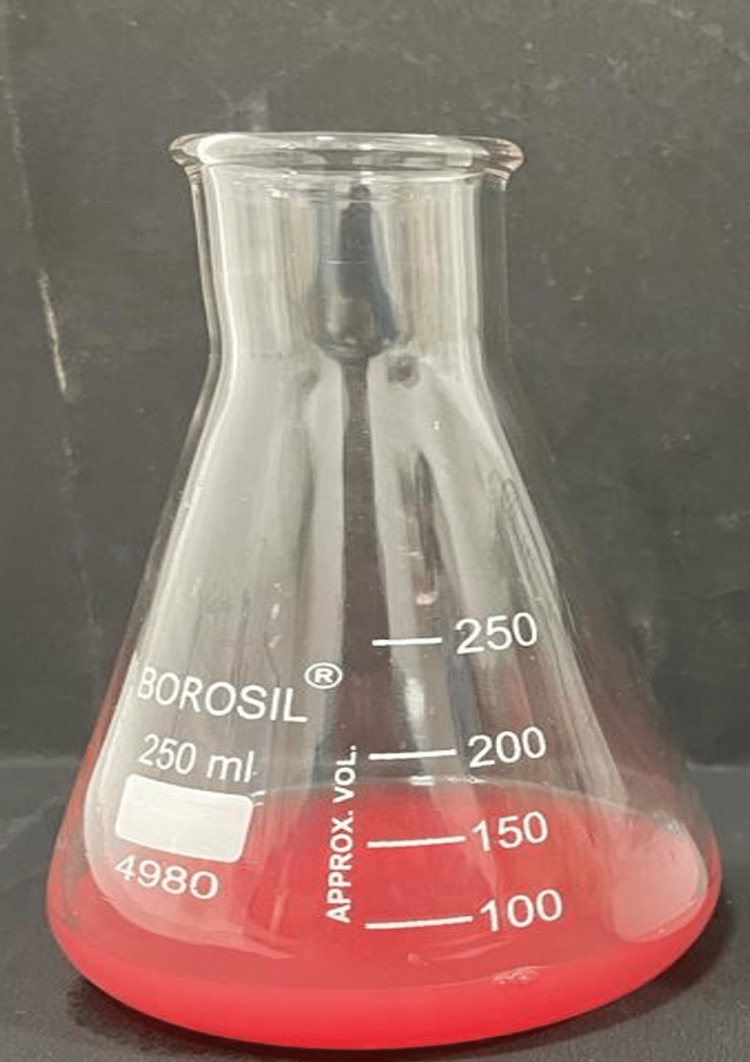
Aqueous watermelon extract

Using a clean mortar and pestle, the watermelon pieces were ground in order to obtain a fine paste, which was collected in a clean beaker. The dilution of the fine paste was undertaken using 100 mL distilled water and was stirred for 15 minutes to ensure the proper extraction of bioactive compounds from the watermelon [12]. Apart from this, the rind of the watermelon was also dried and prepared in the similar method, as seen in Figure 2. The final mixture obtained was the watermelon aqueous extract (Figure 3).

Preparation of stannous chloride (Cl) nanoparticles

Stannous chloride nanoparticles were synthesized using a green synthesis approach mediated by the watermelon extract.

Preparation of Diluted Stannous Chloride Solution

Stannous chloride powder (0.45 g) was weighed to be added to 60 mL of distilled water. The mixture was stirred until the powder completely dissolved, resulting in a diluted stannous chloride solution.

Addition of Watermelon Extract to Stannous Chloride Solution

**Figure 4:**
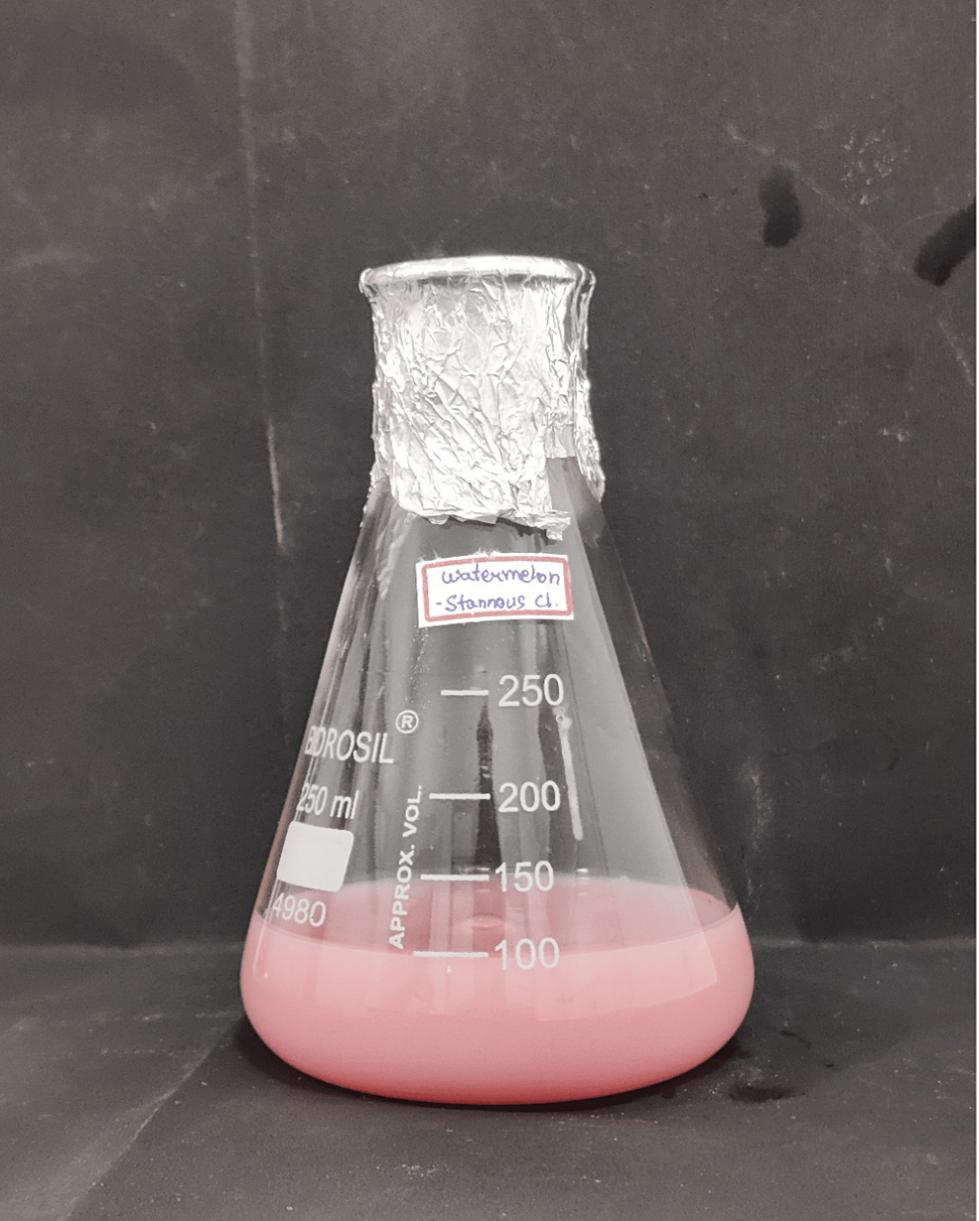
Final solution obtained by mixing stannous chloride solution and aqueous watermelon extract

The diluted stannous chloride solution was transferred to a clean flask. To this solution, 1 mL of the watermelon extract prepared earlier was added. The mixture was stirred gently to ensure the uniform mixing of the components (Figure 4).

Orbital Shaking and Centrifugation

**Figure 5:**
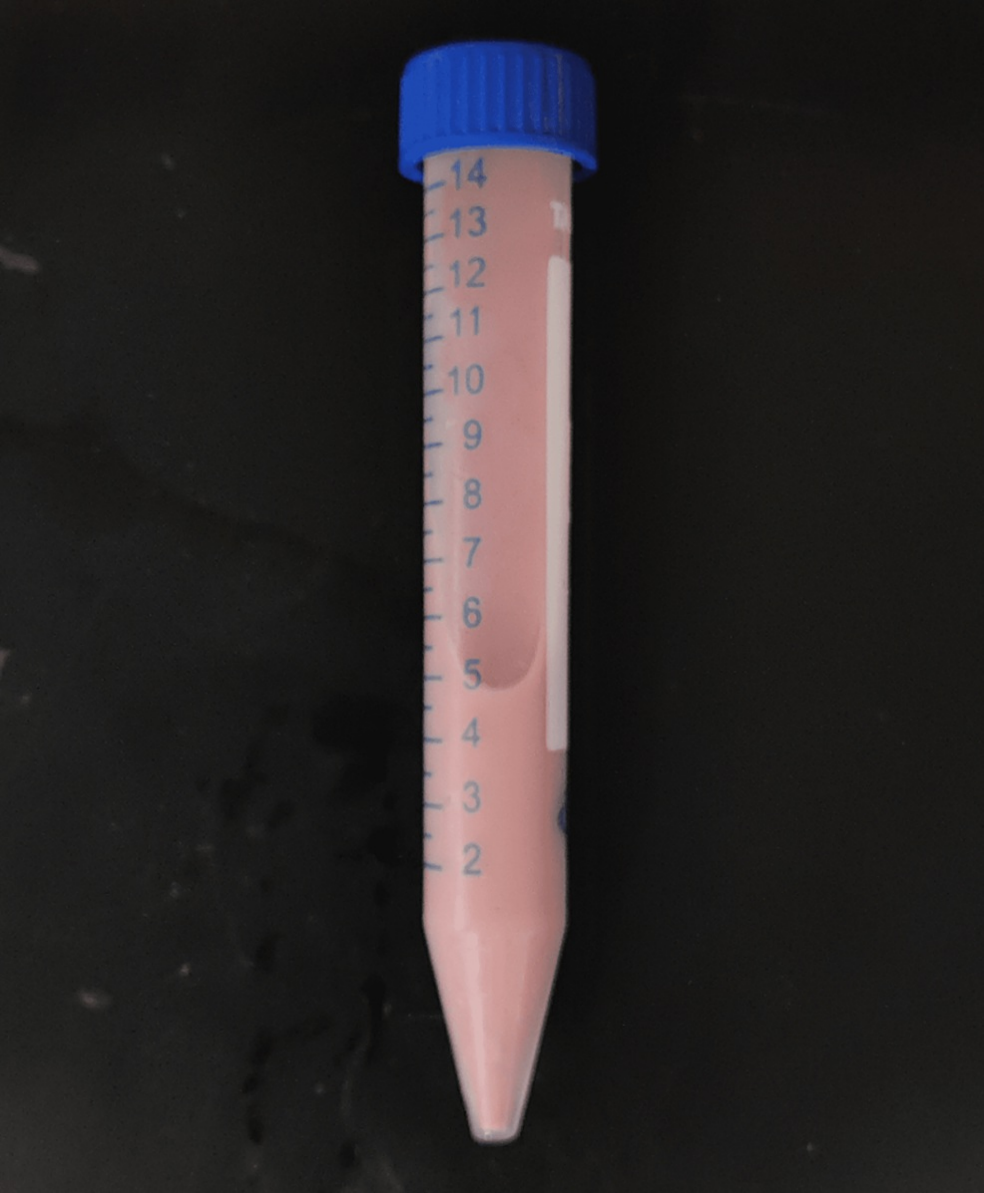
Color change as an indicator of nanoparticle synthesis

The stannous chloride and watermelon extract mixture was subjected to orbital shaking using a laboratory orbital shaker for a specific duration, typically 2-3 hours. The orbital shaking helps in the metal ions to be reduced and subsequent nanoparticle formation. After the completion of the shaking process, the mixture was centrifuged at 8000 rpm for 10 minutes to separate the nanoparticles from the remaining solution [13]. One of the early indicating signs of a reduction of metal salts into nanoparticles is the visual observation of color change in a solution. This is the end point indicator of nanoparticle synthesis, as shown in Figure 5.

Characterization of stannous nanoparticles (SnNPs)

The synthesized stannous chloride nanoparticles were subjected to various characterization techniques to evaluate their properties and determine their effectiveness against *Streptococcus mutans*.

Fourier Transform Infrared (FTIR) Spectroscopy

FTIR (Thermo Nicolet Avatar 330, Thermo Fischer Scientific, Waltham, MA) analysis was performed to identify the functional groups present in the synthesized nanoparticles. The nanoparticles were mixed with potassium bromide (KBr) and then compressed to a pellet for FTIR analysis [14].

Antibacterial Testing

Using the proper microbiological procedures, the nanoparticles' antibacterial activity was assessed against *Streptococcus mutans*. The minimum inhibitory concentration (MIC) and the zone of inhibition were both determined using the broth dilution method or the agar well diffusion method, respectively [15].

Energy-Dispersive X-ray (EDAX) Analysis

EDAX analysis was conducted to determine the elemental composition of the synthesized nanoparticles. The nanoparticles were placed on a suitable substrate and subjected to EDAX analysis using a scanning electron microscope equipped with an EDAX detector (D8 Advance Diffractometer, Bruker Corporatio, Billerica, MA) [16].

Scanning Electron Microscopy (SEM)

SEM was employed to examine the morphology and size of the nanoparticles. The synthesized nanoparticles were deposited on a sample holder, coated with a thin layer of conductive material, and analyzed using a high-resolution scanning electron microscope.

Antimicrobial activity of AgNPs against oral pathogens

Using a Mueller-Hinton agar (MHA) plate, the agar well diffusion method was utilized to assess the antibacterial activity of various doses of AgNPs against oral pathogens such as *Streptococcus mutans*. The MHA was made with double-distilled water (pH 7.0) and sterilized for 15 minutes in an autoclave at 121°C. The sterilized MHA was then poured into the petri plate and let to laminar flow solidify at room temperature. A sterile cotton swab dampened with the suspension of the relevant microbial culture was used to apply an inoculum comprising 106 cfu/mL of the freshly made bacterial culture to the Mueller-Hinton agar (MHA) plates.

With the aid of a micropipette, three wells with a diameter of 9 mm were then drilled into the MHA medium and filled with varying amounts (50 μg, 100 μg, and 125 μg) of the final nanoparticle solution. The solution was then allowed to diffuse into the medium for four hours at room temperature. The culture plates were then kept at 37°C for a further 24 hours. Each plate's zone of inhibition's diameter (mm) was measured after incubation. Apart from this, minimum inhibitory concentration (MIC) was calculated using the broth dilution method. For broth dilution, often determined in 96-well microtiter plate format, microbial agents are inoculated into a liquid growth medium in the presence of different concentrations of an antimicrobial agent. Growth is assessed after incubation for a defined period of time (1-5 hours), and the MIC value is read. This protocol applies only to aerobic bacteria and can be completed in three days [17].

## Results

A color change could occur after the reduction of stannous ions into stannous particles upon contact with plant extracts. Due to the surface plasmon resonance phenomena, stannous nanoparticles exhibit a pale pink tint in aqueous solution, as previously shown in Figure 5. With color shift, SnNP synthesis was verified [18].

Fourier transform infrared (FTIR) spectroscopy

The function of FTIR analysis is the identification of functional groups present in the synthesized stannous chloride nanoparticles mediated by *Citrullus lanatus* extract. The FTIR spectrum showed characteristic peaks corresponding to different functional groups. The presence of peaks at specific wavenumbers confirmed the successful synthesis of the nanoparticles and the involvement of functional groups from the watermelon extract in their formation.

The infrared (IR) spectra of the SnCl_2_ sample show a series of absorption peaks ranging from 800 to 2400. To be more specific, the double peaks ranging from 2339 to 2358 cm^-1^ are strong for the oxygen-carbon (OC) stretching mode of carbon dioxide. The peak at 2005 cm^-1^ is medium for carbon-carbon (CC) stretching mode in the allene group. The peak at 1011 cm^-1^ is strong for the carbon-fluorine (CF) stretching mode of the fluoro compound. The peak at 814 cm^-1^ is strong for carbon-hydrogen (CH) bending.

**Figure 6:**
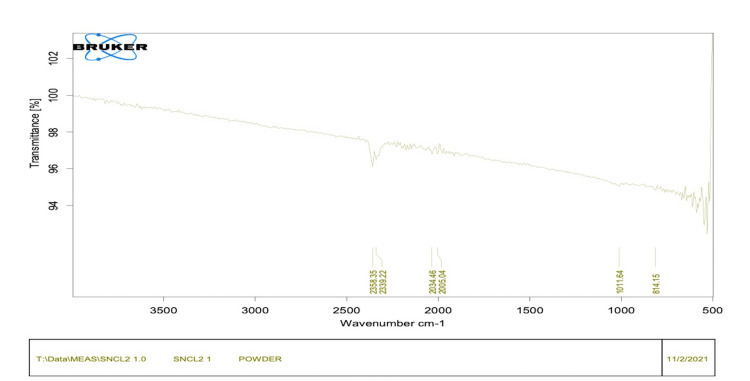
Fourier transform infrared (FTIR) spectroscopy giving peaks in IR spectra IR: infrared

This demonstrates that the plant extract contains reducing groups and secondary metabolites necessary for the formation of biogenic SnNP. Additionally, the Bragg peak's sharp band supports the AgNPs' stability, as shown in Figure 6.

Scanning electron microscopy (SEM)

**Figure 7:**
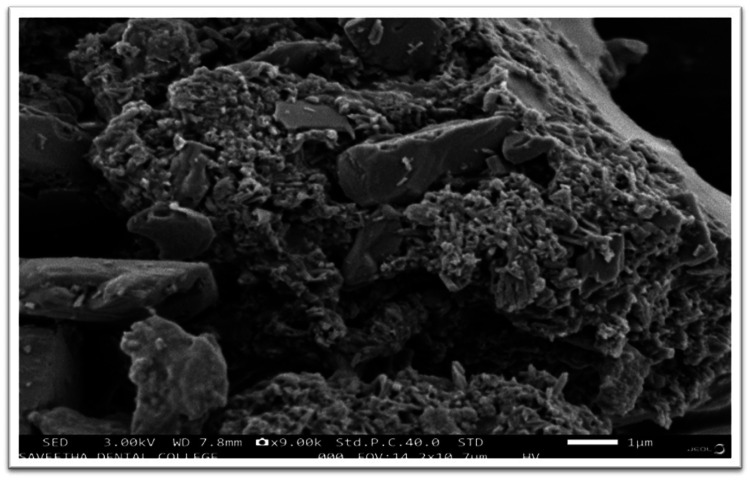
SEM analysis of the stannous nanoparticles SEM: scanning electron microscopy

SEM analysis was performed to examine the morphology and size of the synthesized nanoparticles. The SEM images revealed spherical-shaped nanoparticles with a uniform size distribution. The average particle size was determined by measuring multiple nanoparticles and calculating the mean as 65 nm in size. The SEM analysis provided visual confirmation of the nanoparticle structure and revealed their surface characteristics, as shown in Figure 7.

Energy-dispersive X-ray (EDAX) analysis

To ascertain the elemental makeup of the synthesized nanoparticles, an EDAX analysis was performed. The existence of stannous (Sn) and chloride (Cl) elements was confirmed by the analysis, demonstrating the successful production of stannous chloride nanoparticles (Table 1).

**Table 1:** The results of EDAX analysis EDAX: energy-dispersive X-ray

Element	Percentage
Carbon	49%
Oxygen	30%
Stannous	16%
Chloride	5%

**Figure 8:**
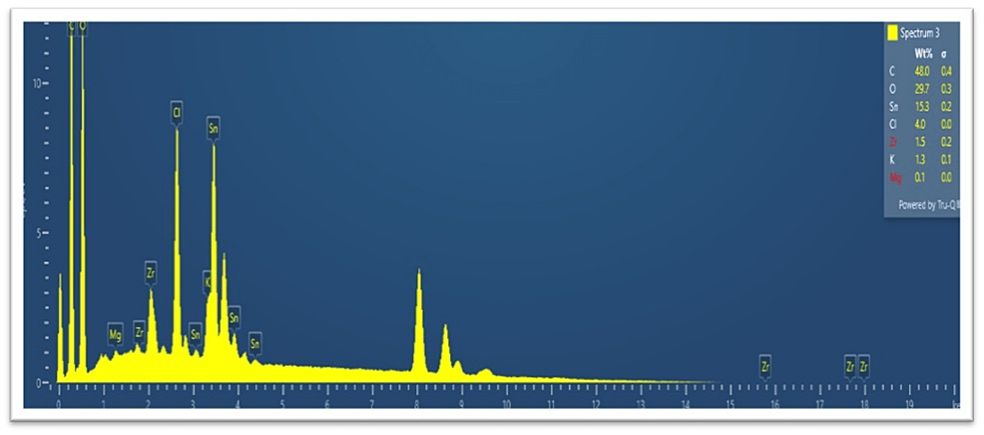
Energy-dispersive X-ray (EDAX) analysis describing the elements detected in the sample with corresponding spectra values

The JEOL JSM 7600F (JEOL USA, Inc., Peabody, MA) was used to conduct the EDAX analysis. The energy-dispersive X-ray (EDAX) analysis spectrum of the nanoparticles is shown along with the elements of oxygen and carbon. It is used to identify chemical compounds that are responsible for the production and stability of nanoparticles, as well as chemical groups that are present in the nanoparticle powder. The peaks were measured at 1096 cm^-1^, as shown in Figure 8.

Antibacterial testing

**Figure 9:**
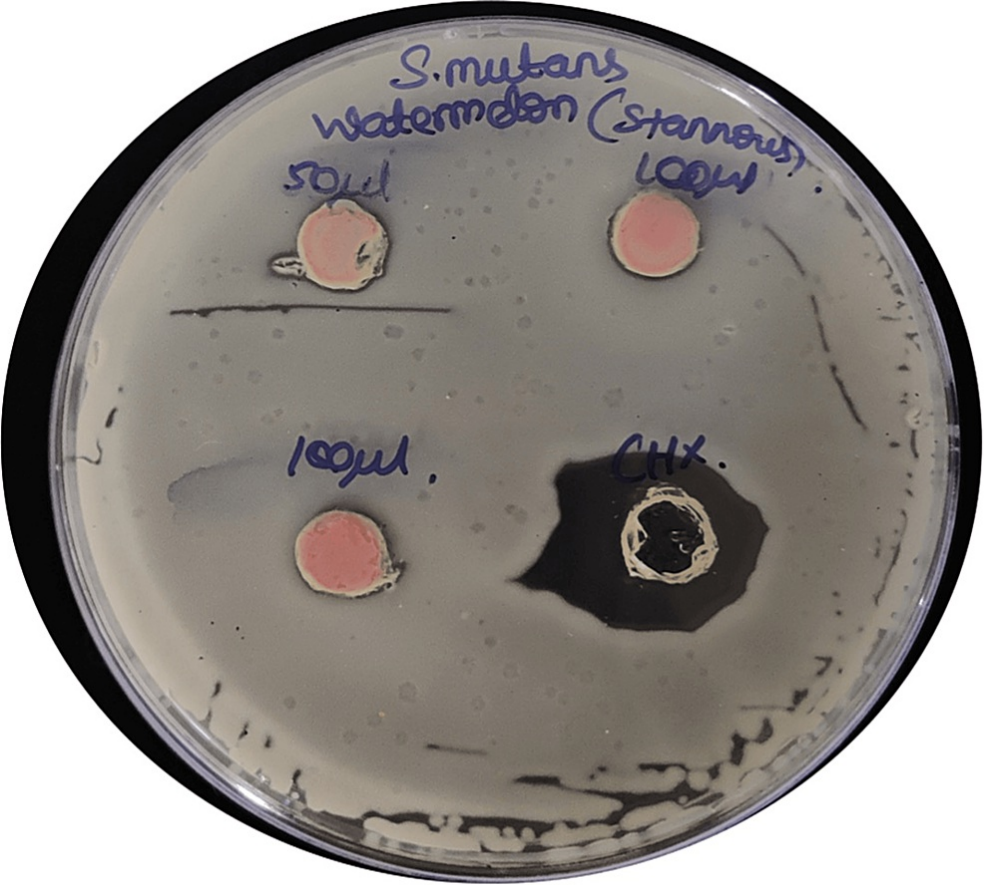
Agar well diffusion method indicating the zone of inhibition around various concentrations of SnNps versus control SnNps: stannous nanoparticles

The antibacterial activity of the synthesized nanoparticles was evaluated against *Streptococcus mutans*. The agar well diffusion method (Figure 9) and the broth dilution method were used to determine the zone of inhibition and minimum inhibitory concentration (MIC), respectively. The results revealed a significant inhibition of *Streptococcus mutans* growth in the presence of the stannous chloride nanoparticles mediated by *Citrullus lanatus* extract (Table 2). The zone of inhibition or MIC values obtained were compared to those of the control group (distilled water) and the positive control group (2% chlorhexidine gluconate). The data presents the MIC values of stannous nanoparticles (SnNPs) at different concentrations and the control (distilled water) over a five-hour time course, as well as the MIC value of chlorhexidine, a commonly used antiseptic. It was seen that at all time points (one hour, two hours, three hours, four hours, and five hours), the MIC values of stannous nanoparticles are generally low, indicating that even relatively low concentrations of SnNPs can inhibit the growth of *Streptococcus mutans*. The MIC values of SnNPs vary with time and concentration. At two hours and three hours, the MIC values show some variation, with higher concentrations showing higher effectiveness against the bacteria compared to the control (distilled water), which consistently has higher MIC values, indicating that it does not inhibit the growth of *Streptococcus mutans* at these concentrations over the given time frame.

**Table 2:** The minimum inhibitory concentration (MIC) of synthesized stannous nanoparticles over various concentrations compared to control SnNP: stannous nanoparticle

Time	50 μL SnNP concentration	100 μL SnNP concentration	150 μL SnNP concentration	Control (distilled water)	Chlorhexidine (100 μL)
1 hour	0.531 μg/mL	0.452 μg/mL	0.753 μg/mL	0.452 μg/mL	0.327 μg/mL
2 hours	0.623 μg/mL	1.322 μg/mL	1.322 μg/mL	0.419 μg/mL	0.845 μg/mL
3 hours	0.699 μg/mL	1.176 μg/mL	1.176 μg/mL	0.477 μg/mL	0.331 μg/mL
4 hours	0.954 μg/mL	0.954 μg/mL	0.954 μg/mL	0.954 μg/mL	0.954 μg/mL
5 hours	0.954 μg/mL	0.954 μg/mL	0.778 μg/mL	0.41 μg/mL	0.778 μg/mL

## Discussion

*Citrullus lanatus*, commonly known as watermelon, is a widely distributed fruit that contains many bioactive compounds such as lycopene, citrulline, flavonoids, and phenolic compounds. The bioactive compounds present in watermelon can contribute to the antibacterial properties and the synthesis of nanoparticles in many ways [19].

Antioxidant and anti-inflammatory properties are present, such as lycopene, vitamin C, and flavonoids. These properties can help combat oxidative stress and inflammation, which are often associated with bacterial infections. By reducing oxidative stress and inflammation, the bioactive compounds can support the body's immune response and potentially inhibit the growth and survival of bacteria [20].

Apart from this indirect action, there is a disruption of bacterial membranes. The compounds can destabilize the lipid bilayer of the bacterial cell membrane, leading to cell lysis and bacterial death. This mechanism of action can be particularly effective against gram-positive bacteria such as *Streptococcus mutans*. One of its other attributes is interference with bacterial adhesion and biofilm formation, making the microorganisms more susceptible to antibacterial treatments.

The bioactive compounds in watermelon can serve as reducing and stabilizing agents in the green synthesis of nanoparticles. For example, the phenolic compounds and citrulline present in watermelon extract can act as reducing agents, facilitating the conversion of metal ions into nanoparticles. Additionally, these compounds can act as stabilizing agents, preventing the agglomeration and subsequent loss of nanoparticle activity. The results of this study demonstrate the successful synthesis of stannous chloride nanoparticles using a green synthesis approach mediated by *Citrullus lanatus* extract. The utilization of watermelon extract as a reducing and stabilizing agent for nanoparticle synthesis offers several advantages, including eco-friendliness, cost-effectiveness, and the potential enhancement of antimicrobial properties. The FTIR analysis confirmed the presence of functional groups derived from the bioactive compounds in the watermelon extract, which played a crucial role in the reduction and stabilization of stannous chloride nanoparticles. The involvement of these functional groups suggests that the nanoparticles may possess additional biological activities attributed to the bioactive compounds present in the watermelon extract.

The antibacterial testing results demonstrated the potent antimicrobial activity of the synthesized stannous chloride nanoparticles against *Streptococcus mutans*. The significant inhibition of bacterial growth observed indicates the potential of these nanoparticles as an alternative antimicrobial agent for combating dental caries caused by *Streptococcus mutans* [21]. The antibacterial efficacy of the nanoparticles can be attributed to their small size, which allows for increased surface area and enhanced interaction with bacterial cells.

The EDAX analysis confirmed the elemental composition of the nanoparticles, with the presence of stannous (Sn) and chloride (Cl) elements. This analysis provided quantitative data regarding the elemental composition and confirmed the successful formation of stannous chloride nanoparticles. The SEM analysis revealed the morphology and size of the synthesized nanoparticles. The spherical shape and uniform size distribution observed in the SEM images indicate the successful synthesis of well-defined nanoparticles. The average particle size, determined from the SEM images, provides valuable information for the further optimization and application of the nanoparticles.

The antimicrobial activity of this study also corresponds to positive results. One hundred milligram of the green synthesized SnNPs was almost equal to 50 mg ampicillin against *S. mutans* bacteria. Since the potent antimicrobial properties of these compounds are well recognized, many researchers are interested in employing other inorganic nanoparticles as antibacterial agents. In comparison to traditional chemical antibacterial treatments, these inorganic nanoparticles have a clear benefit. Multidrug resistance is the main issue brought on by chemical antibacterial agents. Chemical agents' antibacterial properties often depend on how specifically they attach to surfaces and how they are metabolized by microorganisms.

The results suggest that stannous nanoparticles exhibit antibacterial properties against *Streptococcus mutans*, with lower MIC values indicating higher effectiveness. The MIC values appear to vary with both concentration and time, which could be attributed to factors such as nanoparticle uptake by the bacteria, the release of antimicrobial ions, and potential bacterial adaptation.

Comparatively, chlorhexidine demonstrates consistent antibacterial efficacy against *Streptococcus mutans*, as expected from its established use as an antiseptic. The relatively stable MIC values for chlorhexidine throughout the time course suggest its consistent inhibitory effect. However, stannous nanoparticles exhibit time- and concentration-dependent variability, potentially indicating the need for the optimization of nanoparticle concentration and exposure time to achieve consistent antibacterial outcomes. Further investigation into the underlying mechanisms of SnNPs' antibacterial activity and their potential applications in various clinical contexts could provide valuable insights.

One significant limitation of this study is that it is conducted entirely in vitro, which means that the results might not fully reflect the complex interactions that occur in a living organism and they may not accurately predict how the synthesized nanoparticles will behave in an actual biological system. The transition from laboratory conditions to real biological systems can yield different outcomes due to factors such as bioavailability, distribution, metabolism, and excretion.

Apart from this, the study may not explore in depth the mechanisms of nanoparticle uptake by *Streptococcus mutans* or the specific antimicrobial mechanisms at play, which may in turn not provide a comprehensive assessment of nanoparticle dosages and potential toxicity. The study's time frame is also very limited, and potential long-term effects of repeated nanoparticle exposure are not explored. In some cases, nanoparticles might exhibit different behaviors or toxicities over extended exposure periods. Also, the purity of the watermelon extracts cannot be fully quantified as the entire fruit does not contain equal parts of phenols present that are needed as a capping agent.

By acknowledging these limitations, future researchers can be guided toward addressing the lacunae in this area of research and progress toward a more comprehensive understanding of green nanoparticle synthesis for antimicrobial purposes.

To summarize, the antibacterial and green synthesis potential of watermelon bioactive compounds can contribute to the effectiveness of nanoparticles against bacteria such as *Streptococcus mutans* [22]. These compounds can exhibit direct antibacterial effects, interfere with bacterial adhesion and biofilm formation, and perform the vital role of reduction and stabilization agents in the synthesis of nanoparticle. Harnessing the properties of bioactive compounds form various plant and fruit extracts can lead to the development of novel antibacterial agents and sustainable nanoparticle synthesis methods with potential applications in oral health and beyond.

## Conclusions

There is no restriction on the use of plant extracts to make metal NPs, but the yield of NPs made from pure polyphenols is modest. The produced SnNPs had impressive antibacterial activity against the bacterial strain *S. mutans*. The outcome of the study is encouraging, and further clinical research and studies are warranted to confirm the observations seen and to develop these nanoparticles as commercially available products such as toothpaste/medicament for further use.
